# The potential use of digital health technologies in the African context: a systematic review of evidence from Ethiopia

**DOI:** 10.1038/s41746-021-00487-4

**Published:** 2021-08-17

**Authors:** Tsegahun Manyazewal, Yimtubezinash Woldeamanuel, Henry M. Blumberg, Abebaw Fekadu, Vincent C. Marconi

**Affiliations:** 1 Addis Ababa University, College of Health Sciences, Center for Innovative Drug Development and Therapeutic Trials for Africa (CDT-Africa), Addis Ababa, Ethiopia; 2 Emory University School of Medicine and Rollins School of Public Health, Atlanta, GA USA

**Keywords:** Health policy, Translational research

## Abstract

The World Health Organization (WHO) recently put forth a Global Strategy on Digital Health 2020–2025 with several countries having already achieved key milestones. We aimed to understand whether and how digital health technologies (DHTs) are absorbed in Africa, tracking Ethiopia as a key node. We conducted a systematic review, searching PubMed-MEDLINE, Embase, ScienceDirect, African Journals Online, Cochrane Central Registry of Controlled Trials, ClinicalTrials.gov, and the WHO International Clinical Trials Registry Platform databases from inception to 02 February 2021 for studies of any design that investigated the potential of DHTs in clinical or public health practices in Ethiopia. This review was registered with PROSPERO (CRD42021240645) and it was designed to inform our ongoing DHT-enabled randomized controlled trial (RCT) (ClinicalTrials.gov ID: NCT04216420). We found 27,493 potentially relevant citations, among which 52 studies met the inclusion criteria, comprising a total of 596,128 patients, healthy individuals, and healthcare professionals. The studies involved six DHTs: mHealth (29 studies, 574,649 participants); electronic health records (13 studies, 4534 participants); telemedicine (4 studies, 465 participants); cloud-based application (2 studies, 2382 participants); information communication technology (3 studies, 681 participants), and artificial intelligence (1 study, 13,417 participants). The studies targeted six health conditions: maternal and child health (15), infectious diseases (14), non-communicable diseases (3), dermatitis (1), surgery (4), and general health conditions (15). The outcomes of interest were feasibility, usability, willingness or readiness, effectiveness, quality improvement, and knowledge or attitude toward DHTs. Five studies involved RCTs. The analysis showed that although DHTs are a relatively recent phenomenon in Ethiopia, their potential harnessing clinical and public health practices are highly visible. Their adoption and implementation in full capacity require more training, access to better devices such as smartphones, and infrastructure. DHTs hold much promise tackling major clinical and public health backlogs and strengthening the healthcare ecosystem in Ethiopia. More RCTs are needed on emerging DHTs including artificial intelligence, big data, cloud, cybersecurity, telemedicine, and wearable devices to provide robust evidence of their potential use in such settings and to materialize the WHO’s Global Strategy on Digital Health.

## Introduction

Health technology innovations are transforming the discovery, development, and delivery of health products and services^1–4^ and significantly changing the way health conditions are diagnosed, treated, and prevented^5,6^. These innovations are building a sustainable foundation for affordable, accessible, and high-quality medicines, vaccines, medical devices, and system innovations, pursuing novel solutions, entrepreneurial ventures, and public sector efforts to the most challenging health problems^7,8^. Digital health technologies (DHTs)^9–21^, pharmacoginomics^22,23^, and process innovations^24–27^ are rapidly emerging as promising health interventions. More innovations are expected to emerge as healthcare demand and spending rise^28,29^. Nevertheless, many of these breakthroughs have not reached the healthcare providers and the people most in need to tackle the rising burden of diseases^30–32^. People living in low-income countries, such as many countries in Africa, are at high risk of many health conditions compared to those living in other regions, while having the most limited access to health innovations^30,33^. Africa has the greatest healthcare challenges in the world: life expectancy is 60 years, substantially lower than the global average of 72 years; maternal mortality ratio is 547 per 100,000, but 13 in high-income countries and 216 globally; under 5 mortality is 76 per 1000, but 5 in high-income countries and 39 globally^34,35^. While there were 1098 researchers per million inhabitants globally, the corresponding figure for Africa was 87.9 per million^35^. Africa lags in the capacities for health technology innovations, while it bears 23% of the global disease burden and 16% of the world population, with the continent expected to double its population by 2050, from 1 billion to nearly 2.4 billion^36–40^.

Without urgent technological, industrial, intellectual, and research-oriented health interventions, Africa cannot tackle the needs and demands of its population. If health technology innovations are needed to transform health system gaps in Africa, it is important to generate country-specific evidence to identify challenges and opportunities in the region as potential resources for further interventions. The World Health Organization (WHO) embraced a more proactive stance in this regard. In 2020, the WHO developed a global strategy on digital health for 2020–2025^41^. The vision of the strategy was to improve health for everyone, everywhere by accelerating the development and adoption of appropriate, accessible, affordable, scalable, and sustainable person-centric digital health solutions to prevent, detect and respond to epidemics and pandemics, developing infrastructure and applications that enable countries to use data to promote health and wellbeing, and to achieve the health-related United Nations’s Sustainable Development Goals (SDGs). Through its Africa office, the WHO Regional Office for Africa (WHO-AFRO), the WHO designed the health technologies and innovations program to guide the assessments, development, ethics, use, and monitoring of national health technology strategies, with a broader aim of improving access, quality, and rational use of health innovations, including medicines, medical products, and technologies^42^. Similarly, in 2019, the WHO developed a guideline that established recommendations on DHTs for health systems.

This study is in support of the WHO’s DHT initiatives. We focused on Ethiopia, the fastest growing economy in Africa per the 2019 World Bank report^43^, and the second most populated country, with more than 117 million people in 2021. Ethiopia aims to reach lower-middle-income status by 2025, with strong commitment and dedication to achieve the SDGs by 2030. The Ethiopian Ministry of Health (MOH) recently, on 06 August 2020, launched a Digital Health Innovation and Learning Center, the first of its kind, where experts can design and validate digital health tools, synthesize and promote best practices, and scale-up innovations^44^. As of 30 April 2021, there were 54.7 million total telecommunication subscribers in the country^45^. Mobile voice subscribers reached 52.8 million, data and internet users 25 million, fixed broadband subscribers 349,000 and fixed service subscribers 923,000. The telecom population and geographic coverage reached 95% and 85.4%, respectively and the density reached 50%^45^. On 22 May 2021, the Ethiopian government awarded a new nationwide telecom license to the Safaricom-led consortium that includes its parent firms Vodafone and Vodacom, British development finance agency CDC Group and Japan’s Sumitomo Corporation after submitting a financial bid offering US$850 million^46^. The Consortia is expected to invest over $8 billion and create jobs for US$1.5 million citizens. The Ethiopian Health Sector Transformation Plan recognizes the need for improving digital health infrastructure to facilitate equitable access to quality healthcare for all Ethiopians^47^. A systematic review of digital health technology-enabled research in Ethiopia has not been synthesized to inform debates and decisions.

Thus, we aimed to investigate whether and how digital health technologies (DHTs) are absorbed in Africa, tracking Ethiopia as a key node, through a systematic review of available studies.

## Results

### Characteristics of included studies

**Fig. 1:**
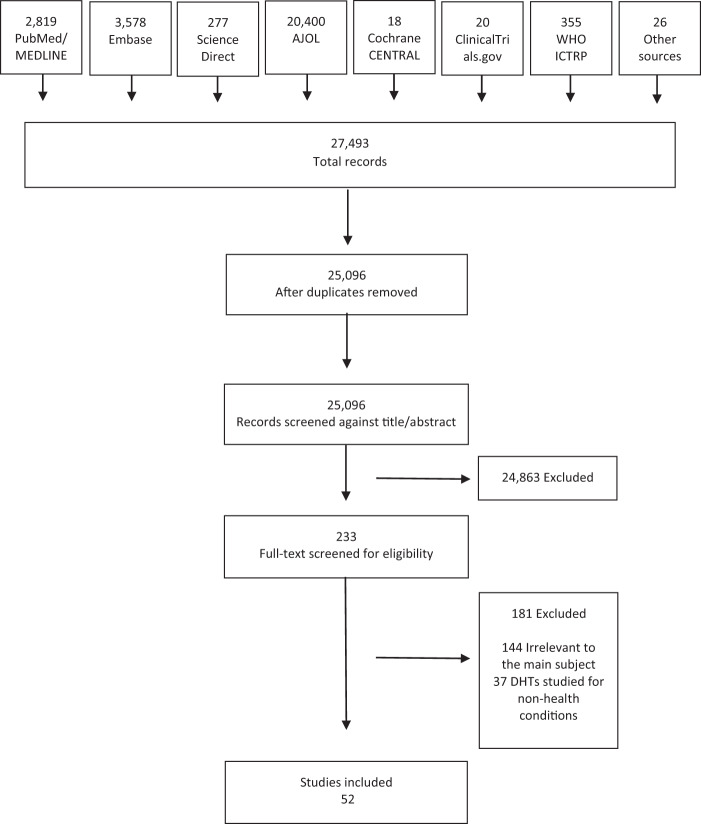
PRISMA flow diagram of the study. PRISMA (preferred reporting items for systematic reviews and meta-analyses) flow diagram of included studies.

Study selection: From the 27,493 articles screened, 2397 duplicates were removed and 24,863 were excluded based on the title or abstract. The rest of 233 full-text articles were screened for eligibility, of which 181 were excluded for being irrelevant to the main subject (144) or focused on non-health conditions (37). Fifty-two (52) studies were identified that met the inclusion criteria. Figure 1 summarizes the PRISMA flowchart of the study.

Participants: The 52 included studies had a total of 596,128 study participants. The health conditions studied were maternal and child health, including antenatal care, postnatal care, infant feeding, contraceptives, and delivery (*n* = 15), infectious diseases including TB, HIV, malaria, lymphatic filariasis, and onchocerciasis (*n* = 14), non-communicable diseases including diabetes and cancer (*n* = 3), dermatitis (*n* = 1), surgery (*n* = 4), and the remaining 15 had general health conditions.

Interventions: The included studies were involved six digital health domains: 29 on mobile health (mHealth) (574,649 participants); 13 on EHR (4534 participants); four on telemedicine (465 participants); two on Cloud usage (2382 participants); three on information and communication technology (ICT, 681 participants), and one on AI (13,417 participants). Our search did not find articles relevant to wearable devices, software as a medical device, computing sciences in big data, cybersecurity, wireless medical devices, and robotics.

Outcomes: The primary outcome variables were feasibility (*n* = 12), usability (*n* = 9), willingness or readiness to use (*n* = 10), effectiveness (*n* = 10), quality improvement (*n* = 7), and knowledge or attitude about the DHT (*n* = 4). Some of the studies employed two or more of these outcomes.

Study type: The study designs were cross-sectional study (*n* = 41), RCT or non-randomized experimental (6), and cohort (5).

Study setting: All the studies were conducted in Ethiopia. Some of the studies were multi-country with a significant number of participants included from Ethiopia.

### mHealth

There were 29 publications identified in the mHealth field^48–76^, involving a total of 574,649 study participants: 402,542 patients, 171,295 healthy individuals, and 812 healthcare professionals. The most common citations were on the potential use of mHealth for maternal, child, and reproductive health, covering 52% (15/29) of the citations, followed by infectious diseases, 48% (14/29). The publications were mainly cross-sectional, 69% (20/29), followed by RCT, 17% (5/29). The outcomes of interest were effectiveness (*n* = 9), usability (*n* = 5), feasibility (*n* = 6), quality (*n* = 4), willingness (*n* = 4), and knowledge (*n* = 1). Table 1 summarizes the characteristics of the included mHealth studies.

**Table 1:** Characteristics of included mHealth studies (*n* = 29).

Reference	mHealth	Condition	Participants type	Participants #	Study design	Outcome measure	Finding
Yigezu et al.^48^	Mobile-based VCT	HIV VCT	VCT attendants	144,267	Cross-sectional - cost-effectiveness	Effectiveness—cost-effectiveness	Mobile-based VCT costs less than both facility-based and stand-alone VCTs
Gebremariam et al.^49^	SMS	Infant feeding	Parents of child-bearing age	41	Cross-sectional	Feasibility, acceptability	Feasible and acceptable option for knowledge sharing and awareness
Starr et al.^50^	mHealth	Post-surgery follow-up	Patients on post-surgery follow-up	701	Cohort, prospective	Feasibility	Telephone follow-up after surgery is feasible and valuable
Jadhav et al.^51^	Own mobile phone	Contraceptive	Women of reproductive age	15,683	Cross-sectional, retrospective	Effectiveness— Contraceptive uptake	No association between mobile phone ownership and contraceptive uptake
Bradley et al.^52^	Smartphone	Post-surgery follow-up	Patients on post-surgery follow-up	24	Cohort	Feasibility	smartphones were low-cost, reliable method to follow-up patient after surgery
Nesemann et al.^53^	Smartphone-CellScope device for conjunctival photograph	Trachomatous inflammation - follicular	Children aged 1–9 yrs	412	Cross-sectional	Effectiveness	84.1% sensitive 97.6% specific
Tadesse et al.^54^	mHealth- based e-Partograph	Obstetric care	Healthcare professionals	466	Cross-sectional	Willingness	46% willing to use mobile-phone for e-Partograph
Kassa et al.^55^	Own mobile phone	Postnatal care	Women in postnatal care	370	Cross-sectional	Knowledge, attitude	3x higher odds of positive attitude to preconception in women who own phone
Kebede et al.^56^	SMS or voice call reminder	Postnatal appointment	Women in postnatal care	700	RCT	Effectiveness— Postnatal compliance	3x higher odds of postnatal compliance in women who received a reminder
Thomsen et al.^57^	mHealth-based Safe Delivery App	Delivery	Healthcare professionals	56	Cross-sectional	Usability—user experience	The App improved providers’ delivery knowledge and skills
Jemere et al.^58^	mHealth-based health services	Diabetes	Patients with diabetes	423	Cross-sectional	Willingness, access,	78% had a phone; 71% willing to receive mHealth-based diabetes services
Habtamu et al.^59^	Smartphone-based Contrast Sensitivity Test ((PeekCS)	Contrast Sensitivity (CS)	Adults with trachomatous trichiasis	147	RCT	Effectiveness	It is repeatable, rapid, accessible and easy to perform CS testing.
Endebu et al.^60^	SMS to support medication adherence	HIV/AIDS	people living with HIV/AIDS receiving antiretroviral treatment	420	Cross-sectional	Feasibility, acceptability	High (90.9%) acceptability of SMS on adherence to antiretroviral therapy
Quinonez et al.^61^	MiGene Family History App	Medical genetics services	Healthcare professionals	47	Cross-sectional	Feasibility	The App was useful for the collection and analysis of genetics data.
Endehabtu et al.^62^	SMS-based intervention	Antenatal care	Women in antenatal care	416	Cross-sectional	Willingness access,	36% had smartphones; 71% willing to receive SMS-based antenatal care intervention
Mengesha et al.^63^	mHealth-based HMIS	Data use	Health extension workers	62	Cross-sectional	Data quality, user experience	mHealth-based HMIS improved data quality, data flow, patient follow-up.
Steege et al.^64^	mHealth-based data and reminder	TB	Health extension workers	19	Cross-sectional	Quality—healthcare delivery	Improved community TB and maternal health service delivery
Martindale et al.^65^	MeasureSMS- morbidity reporting tool	lymphatic filariasis, podoconiosis	Healthcare professionals	59	Cross-sectional, comparative	Effectiveness, cost, time	MeasureSMS tool was more effective, 13.7% less costly than paper-based reporting
Abate et al.^66^	Telepathology Acquiring microscopic images using a smartphone camera	blood cell count, malaria lab diagnosis	Healthcare professionals	2	Cross-sectional	Usability, accuracy	It was fast, cost-effective, and accurate in low resource setting.
Shiferaw et al.^67^	mHealth-based data collection	Maternal health service	Healthcare professionals	15	Experimental/ Implementation	Effectiveness	Timely and complete maternal health data
Atnafu et al.^68^	SMS-based data exchange Ap.	Antenatal care	Women on antenatal care	3240	RCT	Effectiveness—MCH outcomes	9% increased deliveries attended by skilled health workers
Mableson et al.^69^	MeasureSMS-Morbidity reporting tool	Lymphatic filariasis (LF) case estimate	People with LF clinical manifestations	400,000	Cross-sectional	Usability as a reporting tool	The tool improved survey and reporting of clinical burden of LF
Medhanyie et al.^70^	Smartphones for collecting patient data	Maternal health records	Healthcare professional	25	Cross-sectional	Usability	8% improved data completeness compered with paper records
Shiferaw et al.^71^	Locally customized mHealth App.	Delivery and postnatal care	Women on ANC	2261	Cohort	Quality—ANC services utilization	The App improved delivery in health centers, but not ANC visits
Lund et al.^72^	mHealth safe delivery App (SDA)	Perinatal and neonatal survival	Women in active labor, provider	3777	RCT	Quality—Perinatal mortality	The SDA nonsignificantly lowered perinatal mortality compared with standard
Kebede et al.^73^	SMS medication reminders	HIV	HIV patients on ART	415	Cross-sectional	Willingness, access	76% owned cellphone 50.9% willing to receive SMS medication reminder
Medhanyie et al.^74^	Smartphone-based data records	Maternal health	Healthcare professionals	24	Cross-sectional	Usability	The records were useful for day-to-day maternal healthcare services delivery
Desta et al.^75^	mVedio for behavior change	Maternal and newborn health	Community members	540	Cross-sectional.	Effectiveness— Community behavior change	mViedo changed community behavior change on maternal and newborn health in rural Ethiopia
Little et al.^76^	Smartphone open-source health App.	maternal health	Healthcare professionals	37	Cohort	Feasibility—Technical needs	Ownership and empowerment are prerequisites for a successful mHealth program

*ANC* antenatal care, *HMIS* health management information systems, *LF* lymphatic filariasis, *MCH* maternal and child health, *RCT* randomized controlled trial, *SDA* safe delivery app., *SMS* short message service, *VCT* voluntary counseling and testing.

The studies had emerging insights into the potential use of mHealth to transform healthcare and improve access to services by addressing financial, social, or geographic factors, though the findings were not consistent. In a study that compared the cost-effectiveness of facility-based, stand-alone, and mobile-based HIV voluntary counseling and testing, the results revealed a cost-effective and improved VCT service with the use of mobile phones when compared with the two arms^48^. A study that assessed the potential of telephone calls to identify and follow-up post-surgery infections and total complications reported the telephone calls were feasible and valuable^50^. A similar study that assessed the potential of cell phones to follow-up patients after short-term surgical missions found the tool as cost-effective and reliable^52^. The use of SMS-based education was found to be a good option for improving the knowledge and awareness of parents regarding infant feeding^49^.

Several studies assessed the potential of using mHealth for family planning and maternal and child health services. One study reported that a high proportion of pregnant women in an antenatal care clinic had a mobile phone and were willing to receive an SMS text message-based mHealth intervention, though two-third of the participants lacked smartphones to upload some application software^62^. Locally customized mHealth applications during antenatal care significantly improved delivery and postnatal care service utilization through positively influencing the behavior of health workers and their clients^71^. Mobile phone reminders were effective in terms of enhancing adherence to postnatal care appointments, with the potential improving postnatal appointment adherence^69^. On the contrary, a retrospective analysis of demographic and health survey data reported that mobile phone ownership or receiving family planning information via SMS had no significant effect on improving contraceptive uptake^51^.

A study that assessed the potential of mHealth on antiretroviral (ART) services for people with HIV reported that the willingness of patients to receive SMS in support of medication reminders was not consistent, with 49% unwilling to receive the reminders^73^. Age, educational status, and previous experience using the internet had a significant association with their willingness^73^. A cross-sectional study of patients with diabetes showed that a high proportion of the patients had access to mobile phones and were willing to use them for medication reminders^58^.

Ten of the 29 included studies on mHealth had “healthcare professionals” as their target participants. Several of these studies revealed that mHealth had the potential to improve skills and competency of healthcare providers toward safe birth^57^, data quality and flow^63^, patient follow-up^63^, community-based tuberculosis and maternal health service delivery^64^, quality and cost-effective reporting of lymphatic filariasis, and podoconiosis data^65^, and timely and complete reporting of maternal health data^67,70,74,76^. Some healthcare providers were able to appropriately use mHealth technologies for patient assessment and routine data collection with minimal training and supervision. However, there were major preconditions needed for the healthcare providers to effectively apply such technologies, including in-service application training^54^, strong connectivity and electric power supply, especially in rural areas of the country^63^, and uninterrupted mobile network airtime^74^. One study reported on genomics data^61^. A psychiatric genomics consortium exchanged data between research groups for genome-wide association studies on neurogenetics of schizophrenia, with tens of thousands of patients and controls included. The consortium developed and used an mHealth application, MiGene Family History App (MFHA), to assist clinicians with the collection and analysis of patient genetic data over six months and assessed its feasibility through a survey of 47 clinicians. The results showed the potential expansion of medical genetics services into low and middle-income countries (LMICs) and the feasibility and benefit of the MFHA for the services^61^.

### Electronic health records

Of the final set of 52 studies, 13 were on EHR^77–89^, involving 4534 study participants: 4232 health professionals, 250 patients, and 52 healthy individuals. The studies used health information technology, health management information system (HMIS), tablet-based electronic data capture (EDC), electronic information source (EIS), health smart card (HSC), and Android-based data collection system as their EHR of interest. Five studies focus on infectious diseases, one on non-communicable diseases (diabetes), and the remaining seven had a broader health system scope. The studies were all cross-sectional, 92% (12/13), except for one RCT. The outcomes of interest were willingness (*n* = 5), usability (*n* = 3), quality (*n* = 3), and feasibility (*n* = 2). Table 2 summarizes the characteristics of the included EHR studies.

**Table 2:** Characteristics of included EHR studies (*n* = 13).

Reference	EMR	Condition	Participants type	Participants #	Study design	Outcome measure	Finding
Seboka et al.^77^	Information system for managing diabetes	Diabetes	Healthcare professionals	406	Cross-sectional	Willingness, attitude,	64% had a favorable attitude to remotely monitor diabetes patients,74% willing to use voice calls.
Berihun et al.^78^	EMR in health facilities	HIV	Healthcare professionals	616	Cross-sectional	Willingness	86% willing to use EMR
Ahmed et al.^79^	EMR in health facilities	–	Healthcare professionals	420	Cross-sectional	Willingness iIntention	40% intention to use EMR
Kebede et al.^80^	HMIS in health facilities	–	Healthcare professionals	332	Cross-sectional	Quality	48% accuracy and 82% completeness of data; below national standards
Awol et al.^81^	EMR in health facilities	–	Healthcare professionals	414	Cross-sectional	Willingness— readiness	62% ready to use EMR system
Zeleke et al.^82^	Electronic data capture (EDC)- tablet	–	Interviewers	12	RCT	Quality of data	Better data quality and efficiency with EDC than standard paper-based data
Abiy et al.^83^	EMR at ART clinic	HIV	Patients on HIV care	250	Cross-sectional, comparative	Quality— completeness, reliability	Slightly lower (76%) data completeness in EMR, than paper-based (78%)
Bramo et al.^84^	Electronic information sourse (EIS)	HIV/AIDS Care and Treatment	Healthcare professionals	352	Cross-sectional	Usability—utilization	67% not used EIS for not having training, prefer print resource
Dusabe-Richards et al.^85^	HMIS	TB	Healthcare professionals	90	Cross-sectional	Feasibility	HMIS is usable, but with gaps in quality, accuracy, reliability, timeliness of data
Samuel et al.^86^	Electronic Information Sources (EIS)	–	Healthcare professionals	590	Cross-sectional	Usability, access	42% used EIS, affected by computer literacy, access to internet
Tilahun et al.^87^	SmartCard	–	Healthcare professionals	406	Cross-sectional	Usability—user satisfaction,	61% dissatisfied with the EMR; 64% believed EMR had less quality impact
Biruk et al.^80^	EMR		Healthcare professionals	606	Cross-sectional	Willingness—readiness	54% ready to use EMR
King et al.^89^	Android-based data collection	Neglected tropical diseases	Community members (households)	40	cross-sectional, comparative	Feasibility, effectiveness	Suitable, accurate, and save time over standard paper-based survey questionnaires

*EDC* electronic data capture, *EIS* electronic information source, *EMR* electronic medical records, *HMIS* health management information systems, *RCT* randomized controlled trial.

According to the studies reviewed, EHRs have the potential to exchange real-time patient-related data for better clinical decision-making and to capture and share electronic health information efficiently. However, the studies also reported potential gaps and drawbacks associated with EHRs. A study that compared EHR with paper-based records for ART data reported a higher incomplete data with the use of EHR for various reasons including difficulties implementing EHR in high patient load conditions and the frequent need for capturing dual electronic and paper-based data for individual patients^83^.

Studies that assessed the current HMIS practice in healthcare facilities reported several gaps in the accuracy, completeness, and timeliness of data for reasons including poor support from facility management, lack of accountability for data errors, poor supportive supervision, and absence of a dedicated Information Management unit responsible for EHR functions^80,85^.

A significant number of healthcare professionals were either not ready^81,84,88^ or not willing^78,87^ to use EHR. Some healthcare professionals still preferred paper-based records to EHR in their daily work^82,85,87–89^ while other preferred EHR^82^. Lack of access to EHR training, computer skills, and performance expectancy were the major barriers to their willingness or intention to use EHR^77–81,83,84,87,88^.

### Telemedicine

Of the 52 studies considered, four reported on telemedicine, involving 465 participants who were healthcare professionals^90–93^. The studies investigated what level of knowledge and attitude healthcare professionals have toward telemedicine^90^, why healthcare providers resist using telemedicine^91^, and how telemedicine and teledermatology systems can be improved in a given program^92,93^. All were cross-sectional and their outcomes of interest were knowledge and attitude, willingness, usability, and feasibility (Table 3).

**Table 3:** Characteristics of included telemedicine studies (*n* = 4).

Reference	Telemedicine	Condition	Participants type	Participants #	Study design	Outcome measure	Finding
Biruk et al.^90^	Telemedicine	–	Healthcare professionals	312	Cross-sectional	Knowledge, attitude	62% lack good knowledge, 36% lack a good attitude toward telemedicine
Xue et al.^91^	Telemedicine	–	Healthcare professionals	107	Cross-sectional	Willingness -reasons for resistance to telemedicine	Reduced autonomy, anxiety, and costs increased resistance
Delaigue et al.^92^	Teledermatology	Dermatitis	Healthcare professionals	26	Cross-sectional	Usability	Teledermatology delivered a useful service, system gap for case follow-up
Shiferaw et a.^93^	Telemedicine	–	Healthcare professionals	20	Cross-sectional	Feasibility	Telemedicine is in a premature phase and its success needs technology, e-governance, an enabling policy, and multi-sectorial involvement

The studies highlighted that telemedicine has the potential improving healthcare; however, healthcare providers had less knowledge and information about it. One study reported that of the 312 healthcare professionals included, 62% lacked good knowledge and 36% lacked a good attitude about telemedicine^90^. Healthcare professionals resisted the use of telemedicine in their clinical practices mainly due to their perceived threat and controllability, with reduced autonomy, anxiety, and costs indirectly aggravating the resistance^91^. Telemedicine implementation in Ethiopia is influenced by technological dynamics, e-government preparedness, enabling policy environment, multi-stakeholder engagement, and capacity building^93^.

### Cloud-based applications

Two studies reported on Cloud-based interventions, involving 2382 participants: 1748 surgical cases^94^ and 634 healthy women on cervical cancer screening^95^. The aims of the studies were on the feasibility of a multicentre Cloud-based peri-operative registry for surgical care^94^ and the feasibility of a cloud-based electronic data system for human papillomavirus (HPV) cervical cancer screening^95^. Table 4 summarizes the characteristics of the included Cloud studies.

**Table 4:** Characteristics of included Cloud-based studies (*n* = 2).

Reference	Cloud	Condition	Participants type	Participants #	Study design	Outcome measure	Finding
N4PCc^94^	Cloud-based peri-operative registry	Surgical care	Surgical cases	1748	Cohort	Feasibility	A successful multicentre digital surgical registry for key surgery performance indicators and evaluation of peri‐operative outcomes.
Jede et al.^95^	Tablet-based data linked to cloud-based IT	Cervical cancer	Women offered genital self-sampling	634	Cross-sectional	Feasibility	Home-based HPV-DNA self-sampling and clinic-based triage assisted by cloud-based technology was feasible in rural Ethiopia

DNA deoxyribonucleic acid, *HPV* human papillomavirus, *IT* information technology.

A Network for Peri-operative Critical care (N4PCc) developed and evaluated a multicentre Cloud-based peri-operative registry in Ethiopia^94^. The authors reported on 1748 consecutive surgical cases for key performance indicators including compliance with the World Health Organization’s Surgical Safety Checklist, adverse events during anesthesia, and surgical site infections. With these, the authors reported a successful multicentre digital surgical registry that can enable the measurement of key performance indicators for surgery and evaluation of peri‐operative outcomes^95^.

One study conducted home-based human papillomavirus (HPV) self-sampling assisted by a Cloud-based electronic data system. The study used an electronic app-based data system with an offline mode function for tablet computers, based on a Cloud solution. The app-based data system showed robust technical functionality, stability, comfort, data accuracy, and ease-of-use by health workers, with no data loss observed. The offline data collection, uploading, and synchronization system were safe and error-free^95^.

### Artificial intelligence

One study evaluated the precision of AI in differentiating between target and implanted intraocular lens (IOL) power in cataract outreach campaigns in Ethiopia^96^. The study applied machine learning (ML) to optimize the IOL inventory and minimize avoidable refractive error in patients from the cataract campaigns (*n* = 13,417). The result indicated good precision, with the ML optimized the implanted intraocular lens inventory and minimized avoidable refractive error (Table 5).

**Table 5:** Characteristics of included artificial intelligence study (*n* = 1).

Reference	Artificial intelligence	Condition	Participants type	Participants #	Study design	Outcome measure	Finding
Brant et al.^96^	Machine learning (ML)	Cataract surgery	Cataract patients with target and implanted intraocular lens	13,417	Cross-sectional	Effectiveness -precision	ML optimized implanted intraocular lens inventory, minimized avoidable refractive error

*ML* machine learning.

### Information communication technology (ICT)

Three studies reported on ICT, involving a total of 681 participants: 551 healthcare professionals^97,98^ and 130 patients^99^ (Table 6). One study^97^ assessed the digital competency of healthcare providers in seven public health centers and found low-level competency, with factors such as sex, educational status, profession type, monthly income, and years of experience were statistically significant predictors. The second study^98^ assessed health professional’s behavioral intention to adopt eHealth systems and revealed that among the different eHealth constructs, healthcare professionals’ attitude toward eHealth had the strongest effect on the intention to use eHealth systems. The third study evaluated the accuracy of a live-streamed video conference over a third-generation (3G) network for consultation of ultrasound scans from a remote location for a variety of pediatric indications and found the method accurate and feasible^99^.

**Table 6:** Characteristics of included ICT studies (*n* = 3).

Reference	ICT	Condition	Participants type	Participants #	Study design	Outcome measure	Finding
Shiferaw et al.^97^	ICT competency	–	Healthcare professionals	167	Cross-sectional	Knowledge— competency	Low basic digital competency level of healthcare providers
Kalayou et al.^98^	eHealth behavior	–	Healthcare professionals	384	Cross-sectional	Attitude— behavioral intention	Attitude toward eHealth showed the strongest effect on the intention to use eHealth systems
Whitney et al.^99^	Live-stream videos conference using 3G network for ultrasound interpretation	Ultrasound scans from trauma, intussusception, hip effusion	Pediatric emergency patients	130	Cross-sectional	Effectiveness	The ICT system is accurate (92%, 81%, and 88%) and feasible for consultation of ultrasoundscans from a remote location

*3G* third-generation cellular data technology, *ICT* information and communication technology.

## Discussion

We conducted a systematic review of the available literature to provide strong evidence on the potential impact of DHTs on clinical and public health practices in the context of a resource-constrained sub-Saharan African country, Ethiopia. The review identified 52 studies across different areas of DHTs, including mHealth, EMR, telemedicine, cloud-based technology, ICT, and AI. Of the 52 included studies, emerging DHTs had a very small share at 13%: AI 2%, Cloud-based technology 4%, and telemedicine 8%, while the major 81% share was for mHealth (56%), EHR (25%), and ICT 6%). This analysis demonstrated that only 10% (5/52) of the studies were tested in RCTs to provide robust and more credible evidence of the potential of the DHTs. Digital health solutions have substantial benefits and considerable potential to transform the healthcare system and societal wellbeing in Ethiopia. However, their adoption and implementation in full capacity face challenges in terms of infrastructure, training, access to better devices such as smartphones, and some hesitancies from patients and providers. Such challenges have been reported in studies from other African countries including Uganda^100,101^, Kenya^102–105^, and Tanzania^106,107^. A meta-analysis was not conducted due to the heterogeneous nature of the compiled studies.

The mHealth solutions identified in this systematic review mainly aimed to improve maternal and child healthcare and services. This review found that mHealth interventions, either a phone call or SMS, were feasible and acceptable for improving contraceptive uptake, maternal healthcare, and tuberculosis medication adherence among the Ethiopian population. The evidence also showed the potential utility of mHealth for HIV counseling and testing, outpatient follow-up, post-surgery follow-up, child-immunization follow-up, pregnant women antenatal and postnatal follow-ups, and in improving knowledge and awareness of parents regarding infant feeding, while its potential for contraceptive uptake was not significant. The included studies revealed that a significant number of study participants owned mobile phones and were willing to participate in mHealth-related clinical or public health interventions. However, the type of mobile phone that the patients own may not be smartphones to support an upload of needed software. Such challenges were also reported elsewhere in Kenya^108–110^ and Tanzania^111^ where there exist similar socioeconomic disparities in mobile phone ownership in support of the implementation of mHealth.

Our analysis indicates that patients with HIV may resist having their ART adherence information followed up using electronic medication reminders for fear of potential disclosure of their HIV status. The finding was consistent with a recent study in Tanzania that fear for potential involuntary disclosure of HIV status significantly affects mHealth interventions in such patients^112^. The development of the next generation of mHealth in such developing countries requires a broad understanding of the local social contexts that may affect the successes of DHTs^113^. Currently, various innovative DHTs are emerging to address the multifaceted problems associated with tuberculosis diagnosis, care and prevention. However, available data are limited for stronger conclusions of their effectiveness in various countries and settings, including Ethiopia. An RCT is currently ongoing in Ethiopia^114^ to bridge this gap. An initial synthesis of the evidence on DHTs is thus essential to better understand the overall digital health ecosystem in the country and successfully implement DHT-enabled healthcare and research programs.

Our analysis indicates that the use of cloud computing could help resource-constrained countries like Ethiopia to acquire advanced data storage, servers, and databases without investing in new IT infrastructure, though we have identified only two studies that are less likely to support its potential. There have been controversies on the potential benefits of the Cloud and the issues surrounding legal and regulatory implications^115^. For countries like Ethiopia that have not yet established a standardized legal cybersecurity framework, strategy, and governance at the national level^116^, adopting appropriate laws and building technical capacity would reassure the partnership and uptake of Cloud services.

Telemedicine was an emerging technology in the Ethiopian healthcare system which had its drawbacks on successful implementations, despite positive energy that healthcare providers to step up. Building capacity of the healthcare providers before full-scale implementation could bring real benefits out of telemedicine. In the COVID-19 pandemic that restricted physical contacts^117–119^, telemedicine revealed significant contributions in Ethiopia by connecting patients with their healthcare providers to discuss and follow-up their disease conditions^120^.

In recent years, capacities for research, development, and trade on DHTs are rising sharply, while more work is needed to delineate the mechanisms of how the gains could be shared out with resource-constrained countries and global digital health strategy met. Our analysis demonstrated the feasibility and potential demands of DHTs, with the greatest opportunities in emerging health technology markets in Ethiopia.

## Conclusion

DHTs hold much promise tackling major clinical and public health backlogs and strengthening health systems in Ethiopia. Although they are a relatively recent phenomenon in Ethiopia, their potential harnessing clinical and public health practices are highly visible. More RCTs are needed on emerging DHTs including artificial intelligence, big data, cloud, cybersecurity, telemedicine, and wearable devices to provide robust evidence of their potential use in such settings and to materialize the Global Digital Health Strategy.

## Methods

### Study design

This study was based on a systematic review of scientific literature utilizing the Preferred Reporting Items for Systematic Review and Meta-Analysis Protocols (PRISMA-P) 2015 guidelines for the design and reporting of the results. The protocol was registered with PROSPERO (CRD42021240645).

To broaden the scope of DHTs in our review, we combined the latest descriptions of digital health given by the WHO^41^ and the U.S. Drug and Food Administration (FDA)^121^. With this, the following technologies were included in the review: mobile health, telehealth, electronic health records (EHR), telemedicine, health information technology, wearable devices, software as a medical device, artificial intelligence, machine learning, genomics, computing sciences in big data, cybersecurity, wireless medical devices, and medical device interoperability.

### Search strategy

We searched the PubMed-MEDLINE, Embase, ScienceDirect, African Journals Online, Cochrane Central Registry of Controlled Trials, ClinicalTrials.gov, and the WHO International Clinical Trials Registry Platform databases from inception to the latest 02 February 2021 for studies of any design and in any setting in Ethiopia that investigated the potential of DHTs in clinical or public health practices in Ethiopia. We performed manual searches of the WHO website, Google search engine, and reference lists of included studies, and contacted authors of original studies to retrieve extra possible articles or additional data. See the search strategy in the Supplementary information (Supplementary Note 1).

We tailored search strategies to each database and used controlled medical subject headings (MeSHs) and search filters where available, or Boolean search methods and free-text terms, referring to Ethiopia and Digital OR Mobile OR Smartphone OR “Cell phone” OR Techno* OR “short message service” OR SMS OR Tele* OR Telemedicine OR Telehealth OR E-health OR eHealth OR Remote OR Electro* OR Comput* OR cloud OR Software OR Application OR Robotics OR Blockchain OR “Artificial intelligence” OR genomics OR “big data” OR cybersecurity OR wireless.

### Eligibility

Studies were included if they met the following inclusion criteria:

Participants: Eligible participants could be patients, healthcare professionals, data collectors, or healthy individuals in Ethiopia, either women or men, and without age restrictions. Thus, the search was not restricted to participants except that they should reside in Ethiopia.

Interventions: All DHTs that were included in our definition. Studies that investigated digital technologies for non-health conditions were excluded.

Comparisons: Studies with a comparison condition were not required as a criterion. Thus, studies with or without a comparator were eligible.

Outcome: Studies assessing the potential efficacy, effectiveness, feasibility, usability, acceptability, or any related outcomes were included in the review without specific restrictions.

Study design: All available study designs were included. We excluded reviews, commentaries, editorials, and proceedings as these are non‐empirical publications.

### Study selection

Two independent authors examined the title and abstract of all screened publications. From the title and abstract of all publications identified by the database search, those that were duplicated or did not meet the inclusion criteria were excluded. The full texts of the remaining publications were further reviewed. Disagreements were resolved by consensus and, if persisted, were arbitrated through discussion with a third author.

### Data extraction

The identified data were listed, and information was provided on the type and details of the type of DHT under investigation, the disease condition studied, the type of participants, the number of participants, the study design employed, outcome measures, major findings, the surname of the first author, and year of publication.

### Data management and analysis

The publications were grouped in exhaustive tables based on the type of DHT investigated. A qualitative content analysis of all documents and articles was performed. Each article was summarized, and the data were reported descriptively. Less emphasis was placed on the assessment of the quality of the included literature as that was not the major objective of this review.

This analysis was designed to inform our ongoing DHT-enabled randomized controlled trial (RCT) on tuberculosis in Ethiopia (ClinicalTrials.gov, ID: NCT04216420). The trial aims to evaluate the effectiveness of a digital health technology-enabled self-administered therapy over standard directly observed therapy on adherence to TB medication and treatment outcomes in Ethiopia^114^.

### Operational definitions

Mobile health (mHealth): The use of mobile phone device’s core utility of voice or short messaging service (SMS) as well as more complex functionalities to improve health outcomes and healthcare services.

Electronic health records (EHRs): are patient-centered electronic records that provide immediate and secure information to authorized users.

Telemedicine: The practice of medicine at a distance which involves an interaction between a healthcare provider and a patient when the two are separated by distance.

Cloud: The practice of storing and computing health data remotely over the internet, which is managed by external service providers.

Artificial intelligence (AI): The simulation of human intelligence in a digital computer that is programmed to think or perform health tasks like humans.

Information and communications technology (ICT): technologies that provide access to health information through telecommunications.

## Supplementary information

Supplementary Information

## Data Availability

All the data included in this study are available within the paper and its Supplementary Information files.
