# Supplementary material for: The potential use of digital health technologies in the African context: a systematic review of evidence from Ethiopia
Source: NPJ Digit Med. 2021 Aug 17;4:125. doi: 10.1038/s41746-021-00487-4 (PMC8371011; doi:10.1038/s41746-021-00487-4)
Supplement: Supplementary file 1 — Supplementary Information [file 41746_2021_487_MOESM1_ESM.pdf]

Supplementary information

The potential use of digital health technologies in the African context: A systematic review of the evidence from Ethiopia

Supplementary Note 1: Search strategy

1. **PubMed**

S. No	Query	Items found
1	Digital health[MeSH Terms]	1,369
2	Digital[Title/Abstract] OR Mobile[Title/Abstract] OR Smartphone[Title/Abstract] OR "Cell phone"[Title/Abstract] OR Techno*[Title/Abstract] OR "short message service"[Title/Abstract] OR SMS[Title/Abstract] OR Tele*[Title/Abstract] OR Telemedicine[Title/Abstract] OR Telehealth[Title/Abstract] OR E-health[Title/Abstract] OR eHealth[Title/Abstract] OR Remote[Title/Abstract] OR Electro*[Title/Abstract] OR Comput*[Title/Abstract] OR cloud[Title/Abstract] OR Software[Title/Abstract] OR Application[Title/Abstract] OR Robotics[Title/Abstract] OR Blockchain[Title/Abstract] OR "Artificial intelligence"[Title/Abstract] OR genomics[Title/Abstract] OR "big data"[Title/Abstract] OR cybersecurity[Title/Abstract] OR wireless[Title/Abstract]	4,277,229
3	1 OR 2<br>(Digital health[MeSH Terms]) OR (Digital[Title/Abstract] OR Mobile[Title/Abstract] OR Smartphone[Title/Abstract] OR "Cell phone"[Title/Abstract] OR Techno*[Title/Abstract] OR "short message service"[Title/Abstract] OR SMS[Title/Abstract] OR Tele*[Title/Abstract] OR Telemedicine[Title/Abstract] OR Telehealth[Title/Abstract] OR E-health[Title/Abstract] OR eHealth[Title/Abstract] OR Remote[Title/Abstract] OR Electro*[Title/Abstract] OR Comput*[Title/Abstract] OR cloud[Title/Abstract] OR Software[Title/Abstract] OR Application[Title/Abstract] OR Robotics[Title/Abstract] OR Blockchain[Title/Abstract] OR "Artificial intelligence"[Title/Abstract] OR	4,277,413

	genomics[Title/Abstract] OR "big data"[Title/Abstract] OR cybersecurity[Title/Abstract] OR wireless[Title/Abstract])	
4	Ethiopia[Title/Abstract]	17,578
5	3 AND 4<br>((Digital health[MeSH Terms]) OR (Digital[Title/Abstract] OR Mobile[Title/Abstract] OR Smartphone[Title/Abstract] OR "Cell phone"[Title/Abstract] OR Techno*[Title/Abstract] OR "short message service"[Title/Abstract] OR SMS[Title/Abstract] OR Tele*[Title/Abstract] OR Telemedicine[Title/Abstract] OR Telehealth[Title/Abstract] OR E-health[Title/Abstract] OR eHealth[Title/Abstract] OR Remote[Title/Abstract] OR Electro*[Title/Abstract] OR Comput*[Title/Abstract] OR cloud[Title/Abstract] OR Software[Title/Abstract] OR Application[Title/Abstract] OR Robotics[Title/Abstract] OR Blockchain[Title/Abstract] OR "Artificial intelligence"[Title/Abstract] OR genomics[Title/Abstract] OR "big data"[Title/Abstract] OR cybersecurity[Title/Abstract] OR wireless[Title/Abstract])) AND (Ethiopia[Title/Abstract])	2,819

2. ScienceDirect

S. No	Query	Items found
1	Digital OR Mobile OR Smartphone OR "Cell phone" OR Techno OR "short message service" OR SMS OR Tele OR Telemedicine OR Telehealth OR E-health OR eHealth OR Remote OR Electro OR Comput OR cloud OR Software OR Application OR Robotics OR Blockchain OR "Artificial intelligence" OR genomics OR “big data” OR cybersecurity OR wireless AND Ethiopia	277

3. African Journals Online (AJOL)

S. No	Query	Items found
1	Digital OR Mobile OR Smartphone OR "Cell phone" OR Techno OR "short message service" OR SMS OR Tele OR Telemedicine OR Telehealth OR E-health OR eHealth OR Remote OR Electro OR Comput OR cloud OR Software OR Application OR Robotics OR Blockchain OR "Artificial intelligence" OR genomics OR “big data” OR cybersecurity OR wireless AND Ethiopia	20,400

4. **ClinicalTrials.gov**

S. No	Query	Items found
1	Digital OR Mobile OR Smartphone OR "Cell phone" OR Techno OR "short message service" OR SMS OR Tele OR Telemedicine OR Telehealth OR E-health OR eHealth OR Remote OR Electro OR Comput OR cloud OR Software OR Application OR Robotics OR Blockchain OR "Artificial intelligence" OR genomics OR “big data” OR cybersecurity OR wireless AND Ethiopia	20

5. WHO International Clinical Trials Registry Platform (ICTRP)

S. No	Query	Items found
1	Digital OR Mobile OR Smartphone OR "Cell phone" OR Techno OR "short message service" OR SMS OR Tele OR Telemedicine OR Telehealth OR E-health OR eHealth OR Remote OR Electro OR Comput OR cloud OR Software OR Application OR Robotics OR Blockchain OR "Artificial intelligence" OR genomics OR “big data” OR cybersecurity OR wireless AND Ethiopia	355

6. **Embase**

S. No	Query	Items found
1	Digital OR Mobile OR Smartphone OR "Cell phone" OR Techno OR "short message service" OR SMS OR Tele OR Telemedicine OR Telehealth OR E-health OR eHealth OR Remote OR Electro OR Comput OR cloud OR Software OR Application OR Robotics OR Blockchain OR "Artificial intelligence" OR genomics OR “big data” OR cybersecurity OR wireless AND Ethiopia	3,578

7. Cochrane Central Registry of Controlled Trials

S. No	Query	Items found
1	Digital OR Mobile OR Smartphone OR "Cell phone" OR Techno OR "short message service" OR SMS OR Tele OR Telemedicine OR Telehealth OR E-health OR eHealth OR Remote OR Electro OR Comput OR cloud OR Software OR Application OR Robotics OR Blockchain OR "Artificial intelligence" OR genomics OR “big data” OR cybersecurity OR wireless AND Ethiopia	18
